# A personalized time-resolved 3D mesh generative model for unveiling normal heart dynamics

**DOI:** 10.1038/s42256-025-01035-5

**Published:** 2025-05-19

**Authors:** Mengyun Qiao, Kathryn A. McGurk, Shuo Wang, Paul M. Matthews, Declan P. O’Regan, Wenjia Bai

**Affiliations:** 1 Department of Brain Sciences, Imperial College London, London, UK; 2 Data Science Institute, Imperial College London, London, UK; 3 MRC Laboratory of Medical Sciences, Imperial College London, London, UK; 4 National Heart and Lung Institute, Imperial College London, London, UK; 5 Digital Medical Research Center, School of Basic Medical Sciences, Fudan University and Shanghai Key Laboratory of MICCAI, Shanghai, China; 6 UK Dementia Research Institute, Imperial College London, London, UK; 7 Rosalind Franklin Institute, Harwell Science and Innovation Campus, Didcot, UK; 8 Department of Computing, Imperial College London, London, UK

**Keywords:** Cardiovascular diseases, Magnetic resonance imaging

## Abstract

Understanding the structure and motion of the heart is crucial for diagnosing and managing cardiovascular diseases, the leading cause of global death. There is wide variation in cardiac shape and motion patterns, influenced by demographic, anthropometric and disease factors. Unravelling normal patterns of shape and motion, and understanding how each individual deviates from the norm, would facilitate accurate diagnosis and personalized treatment strategies. Here, to this end, we developed a conditional generative model, MeshHeart, to learn the distribution of shape and motion patterns for the left and right ventricles of the heart. To model the high-dimensional spatio-temporal mesh data, MeshHeart uses a geometric encoder to represent cardiac meshes in a latent space and a temporal transformer to model the motion dynamics of latent representations. Based on MeshHeart, we investigate the latent space of 3D + t cardiac mesh sequences and propose a distance metric, latent delta, which quantifies the deviation of a real heart from its personalized normative pattern. Here, 3D + t refers to three-dimensional data evolving over time. In experiments using a large cardiac magnetic resonance image dataset of 38,309 participants from the UK Biobank, MeshHeart demonstrates high performance in cardiac mesh sequence reconstruction and generation. Latent space features are discriminative for cardiac disease classification, whereas latent delta exhibits strong correlations with clinical phenotypes in phenome-wide association studies.

## Abstract (web summary)

MeshHeart, a conditional generative model for time-resolved 3D heart mesh generation, is proposed by Qiao et al. to unravel heart motion patterns. Their findings could advance diagnosis and treatment strategies for cardiovascular diseases.

## Main

The heart is one of the most important and vital organs within the human body^1^. It is composed of four morphologically distinct chambers that function in a coordinated manner. The shape of the heart is governed by genetic and environmental factors^2,3^, as well as a remodelling process observed in response to myocardial infarction, pressure overload and cardiac diseases^4,5^. The motion of the heart follows a periodic nonlinear pattern modulated by the underlying molecular, electrophysiological and biophysical processes^6^. Unveiling the complex patterns of cardiac shape and motion will provide important insights for assessing the status of cardiac health in both clinical diagnosis and cardiovascular research^7–10^.

The current state of the art for assessing cardiac shape and motion is to perform analyses of cardiac images, for example, cardiac magnetic resonance (MR) images, and extract imaging-derived phenotypes of cardiac chambers^9,11^. Most imaging phenotypes, such as chamber volumes or ejection fractions, provide a global and simplistic measure of the complex three-dimensional (3D)–temporal (3D + t) geometry of cardiac chambers^11,12^. However, these global volumetric measures may not fully capture the dynamics and variations of cardiac function across individuals. Recent studies have shown that mesh-based cardiac shape and motion analyses can provide more detailed and clinically relevant insights^13–16^. For example, Piras et al.^14^ proposed to use spatio-temporal motion analysis to identify myocardial infarction. Gilbert et al.^15^ highlighted stronger associations between cardiac risk factors and mesh-derived metrics in the UK Biobank dataset. Mauger et al.^16^ showed that mesh-based motion metrics could independently predict adverse cardiac events. This underscores the importance of establishing a precise computational model of cardiac status to define what a normal heart looks like and moves like. Nevertheless, it is a non-trivial task to describe the normative pattern of the 3D shape or even 3D + t motion of the heart, due to the complexity in representing high-dimensional spatio-temporal data.

Recently, machine learning techniques have received increasing attention for cardiac shape and motion analysis^6,17,18^. Most existing research focuses on developing discriminative machine learning models, that is, training a model to perform classification tasks between different shapes or motion patterns^6,8,19,20^. However, discriminative models offer only classification results and do not explicitly explain what the normative pattern of cardiac shape or motion looks like^21^. By contrast, generative machine learning models provide an alternative route. Generative models are capable of describing distributions of high-dimensional data, such as images^22–24^, geometric shapes^25–27^ or molecules^28,29^, which allow the representation of normative data patterns in the latent space of the model. In terms of generative modelling of the heart, recent developments focus on shape reconstruction and virtual population synthesis^13,30–34^. For example, Xia et al. proposed a method that integrates statistical shape priors with deep learning for four-chamber cardiac shape reconstruction from images^35^. Gaggion et al. introduced HybridVNet, which combines convolutional neural networks with graph convolutions to perform shape reconstruction from multiview images^36^. Dou et al. proposed a conditional flow-based variational autoencoder (VAE) for synthesizing virtual populations of cardiac anatomy^37^ and later developed a compositional generative model for multipart anatomical structures^38^. Beetz et al. introduced a variational mesh autoencoder that models population-wide variations in cardiac shapes with a hierarchical structure^39^ and investigated the interpretability of the latent space extracted from a point-cloud VAE^40^. Although generative models have been explored for cardiac shape reconstruction^35,36^, shape modelling^3,37–39^, image and video generation^41–43^ and data augmentation^44^, their application to personalized normative modelling of the heart from population data remains underexplored.

Here, we provide an endeavour to create a personalized normative model of 3D + t cardiac shape and motion, leveraging deep generative modelling techniques. Cardiac shape and motion are represented by a dynamic sequence of 3D surface meshes across a cardiac cycle. A geometric deep generative model, named MeshHeart, is developed to model the distribution of 3D + t cardiac mesh sequences. MeshHeart uses a graph convolutional network (GCN)^45^ to learn the latent features of the mesh geometry and a transformer to learn the temporal dynamics of the latent features during cardiac motion. This integration enables MeshHeart to model the distributions across both spatial and temporal dimensions. MeshHeart functions as a conditional generative model, accounting for major clinical variables such as sex and age as the generation factor. This enables the model to describe personalized normative patterns, generating synthetic healthy cardiac mesh sequences for a specific patient or a specific subpopulation.

**Fig. 1:**
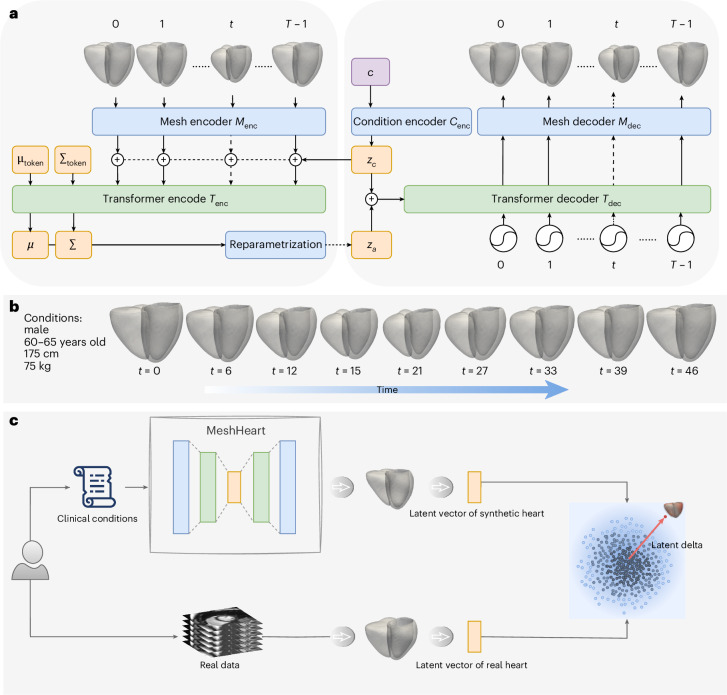
An overview of the MeshHeart model. **a**, Model architecture: MeshHeart encodes a sequence of cardiac meshes using a mesh encoder *M*_enc_ and encodes clinical factors using a conditional encoder *C*_enc_. The encoder outputs across the time frames and along with distribution tokens *μ*_token_ and *Σ*_token_ are processed by a temporal transformer encoder *T*_enc_. A transformer decoder *T*_dec_ and a mesh decoder *M*_dec_ generate a 3D cardiac mesh sequence based on clinical factors. **b**, Given a set of clinical factors, an example of the generated mesh sequence across time frames. **c**, Conceptual framework: MeshHeart constructs a normative cardiac mesh sequence using personal information including age, sex, weight and height. A real heart can be compared with its personalized norm by the latent vector. The latent delta Δ*z* is a distance defined between the latent vector of a synthetic normal heart (dark-blue dot) and that of the real heart (red dot).

We train the proposed generative model, MeshHeart (Fig. 1a), on a large-scale population-level imaging dataset with 38,309 participants from the UK Biobank^9,46^. After training the model, for each individual heart, we can generate a personalized 3D + t cardiac mesh model that describes the normative pattern for this particular subpopulation that has the same clinical factors as the input heart, as shown in Fig. 1c. In qualitative and quantitative experiments, we demonstrate that MeshHeart achieves high accuracy in generating the personalized heart model. Furthermore, we investigate the clinical relevance of the latent vector *z* of the model and propose a distance metric (latent delta Δ*z*), which measures the deviation of the input heart from its personalized normative pattern (Fig. 1c). We demonstrate that the latent vector and latent delta have a highly discriminative value for the disease classification task, and they are associated with a range of clinical features in phenome-wide association studies (PheWAS).

## Results

### MeshHeart learns spatio-temporal mesh characteristics

We first assessed the reconstruction capability of MeshHeart for 3D + t cardiac mesh sequences. The experiments used a dataset of 4,000 test participants, with details of the dataset described in Supplementary Table 1. Each input mesh sequence was encoded into latent representation and then decoded to reconstruct the mesh sequence. Reconstruction performance was evaluated using two metrics, the Hausdorff distance (HD) and the average symmetric surface distance (ASSD), which measure the difference between the input and reconstructed meshes. The HD metric quantifies the maximum distance between points in two sets, highlighting the maximum discrepancy between the original and reconstructed heart meshes. ASSD computes the average distance between the surfaces of two meshes, providing a more holistic evaluation of the model’s accuracy. Evaluation was performed for three anatomical structures: the left ventricle (LV), the myocardium (Myo) and the right ventricle (RV). We compared the performance of MeshHeart with three baseline mesh generative models: Action2Motion^47^, ACTOR^27^ and CHeart^42^. Supplementary Table 2 presents the architecture comparison.

**Fig. 2:**
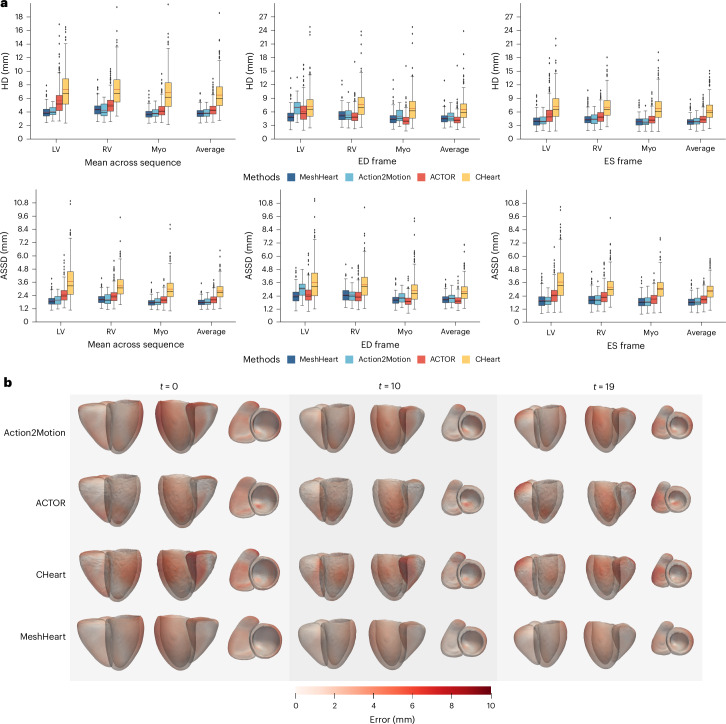
Evaluation of the mesh reconstruction accuracy of MeshHeart, compared with three other methods; Action2Motion, ACTOR and CHeart. **a**, Plots of the HD and ASSD metrics. The metrics are calculated as the mean across all time frames, as well as at the ED frame and the ES frame. They are reported for the LV, the Myo and the RV and averaged across the anatomical structures. Lower values indicate better performance. Each boxplot represents results over *n* = 4,000 UK Biobank participants from the held-out test set, treated as biological replicates. Each participant contributes one sample per method; no technical replicates were used. The unit of analysis is the individual participant. Box plots represent the distribution of the data as follows: the centre line indicates the median; box limits represent the 25th and 75th percentiles (interquartile range); whiskers extend to 1.5× the interquartile range from the box limits; and points beyond this range are plotted individually as outliers. **b**, Visualization of the reconstructed cardiac mesh sequence, coloured by the reconstruction error (in red) between the input mesh and reconstructed mesh. The mesh is visualized in three different imaging planes.

Figure 2a and Supplementary Table 3 report the reconstruction accuracy of MeshHeart, compared with other generative models. The metrics are reported as the average across all time frames, as well as at two representative time frames of cardiac motion: the end-diastolic (ED) frame and the end-systolic (ES) frame. Overall, MeshHeart achieves the best reconstruction accuracy, outperforming other generative models, with the lowest HD of 4.163 mm and ASSD of 1.934 mm averaged across the time frames and across anatomical structures. In addition, Fig. 2b visualizes examples of the reconstructed meshes, with vertex-wise reconstruction errors overlaid, at different frames of the cardiac cycle (*t* (time) = 0, 10 and 19 out of 50 frames in total). MeshHeart achieves lower reconstruction errors compared with the other models and maintains the smoothness of reconstructed meshes. We further conducted ablation studies to assess the contribution of each component to the model performance. These components are described in the Methods, and the detailed results are reported in Supplementary Table 6. Replacing GCN by linear layers results in an increased HD from 4.163 mm to 5.707 mm, while replacing GCN by convolutional neural network results in a HD of 5.268 mm, highlighting GCN’s superiority in encoding mesh geometry. Substituting the transformer with gated recurrent units (GRUs) or long short-term memory networks (LSTMs) leads to an increased HD of 4.720 mm or 5.015 mm, respectively, which demonstrates the advantage of using the transformer for modelling long-range temporal dependencies. Other components such as the smoothness loss term and the distribution parameter tokens also contribute to the model performance. These results highlight MeshHeart’s capability in learning spatial–temporal characteristics of cardiac mesh sequences.

### MeshHeart resembles real data distribution

**Fig. 3:**
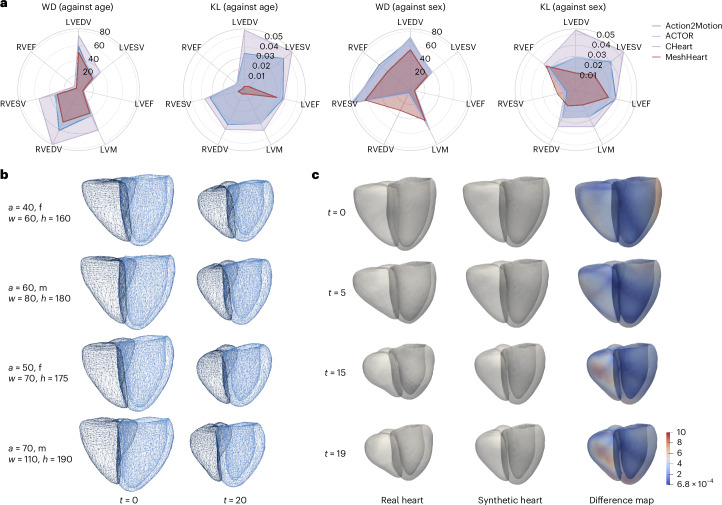
Evaluation of the generation performance of MeshHeart. **a**, Spider charts for the WD and the KL divergence metrics, which quantify the distance between the generated and real data distributions. The data distribution is calculated as the histogram of a cardiac imaging phenotype (LVEDV, LVESV, LVEF, LVM, RVEDV, RVESV and RVEF) against a clinical factor (age or sex). The metrics are plotted over a polar coordinate system, colour-coded by different methods. The smaller the metric (closer to the centre), the greater the similarity between the generated and real data distributions. **b**, Examples of generated 3D + t cardiac meshes with different generating factors, including age (*a*), sex (f and m for female and male, respectively), weight (*w*) and height (*h*). **c**, A side-by-side comparison of a real heart versus the generated synthetic heart and the difference map between them.

Utilizing the latent representations learned by MeshHeart, we assessed the ability of the model to generate new synthetic cardiac mesh sequences that mimic real heart dynamics. To evaluate the fidelity and diversity of the generation, we calculated the similarity between the distributions of real meshes and generated synthetic meshes. For each real heart in the test set (*n* = 4,000), we applied MeshHeart to generate synthetic mesh sequences using the same clinical factors (age, sex, weight and height) as the individual as the model input. During the generation stage, we chose 20 random samples from the Gaussian distribution of the latent space and generated the corresponding mesh sequences. For both real and synthetic meshes, clinically relevant metrics for cardiac structure and function were derived, including left ventricular ED volume (LVEDV), left ventricular ES volume (LVESV), left ventricular ejection fraction (LVEF), left ventricular myocardial mass (LVM), right ventricular ED volume (RVEDV), right ventricular ES volume (RVESV) and right ventricular ejection fraction (RVEF). For each metric *m*, its probability distributions against age *P*(*m*∣*c* = age) and against sex *P*(*m*∣*c* = sex) were calculated. The similarity between real and synthetic probability distributions was quantified using the Kullback–Leibler (KL) divergence^48^ and the Wasserstein distance (WD)^49^, with a lower value denoting a higher similarity, that is, better generation performance. KL divergence is a metric from information theory that evaluates the dissimilarity between two probability mass functions. Similarly, WD measures the dissimilarity between two probability distributions. MeshHeart’s ability to replicate real data distributions is quantitatively demonstrated in Fig. 3a. MeshHeart achieves lower KL and WD scores compared with existing methods, as shown by radar plots with the smallest area, suggesting that the synthetic data generated by the proposed model align closely with the real data distribution for clinically relevant metrics. Supplementary Tables 4 and 5 report the detailed KL divergence and WD scores for different methods.

For qualitative assessment, Fig. 3b shows four instances of synthetic cardiac mesh sequences for different personal factors (age, sex, weight and height). For brevity, only two frames (*t* = 0 and 20) are shown. The figure demonstrates that MeshHeart can mimic authentic cardiac movements, showing contractions across time from diastole to systole. Figure 3c compares a real heart with a synthetic normal heart, at different time frames (*t* = 0, 5, 15 and 19), demonstrating the capability of MeshHeart in replicating both the real cardiac structure as well as typical motion patterns.

We also examined the latent representation learnt by MeshHeart using *t*-distributed stochastic neighbour embedding visualization^50^ as illustrated in Supplementary Fig. 1. The *t*-distributed stochastic neighbour embedding plot projects the 64-dimensional latent representation of a mesh, extracted from the last hidden layer of the transformer encoder *T*_enc_, onto a two-dimensional space, with each point denoting a mesh. It shows ten sample sequences. For each sample, the latent representations of the meshes across time frames form a circular pattern that resembles the rhythmic beating of the heart^51^.

### Latent vector aids cardiovascular disease classification

**Fig. 4:**
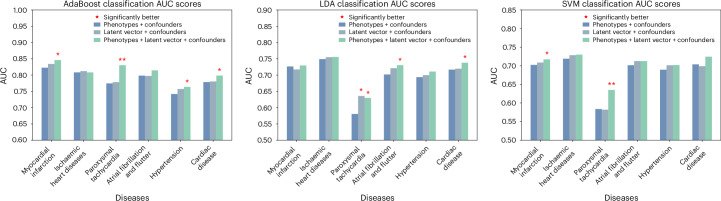
Comparison of disease classification performance, in terms of the AUC scores, when different feature sets are used. The three feature sets being compared include ‘phenotypes + confounders (age, sex, weight, height)’, ‘latent vector + confounders’ and ‘phenotypes + latent vector + confounders’, with each feature set represented by a unique colour in the plot. The three subplots show the performance of three different classifiers, AdaBoost, LDA and SVM. The *x* axis denotes the disease type, and the *y* axis denotes the AUC score. AUC values are averaged across five random train–test splits. For the three feature sets, each feature set is compared with one of the other two features sets using the two-sided DeLong’s test. A single asterisk denotes a notable difference (*P* < 0.05), indicating that a feature set outperforms another feature set markedly, while a double asterisk indicates that a feature set outperforms both of the other two feature sets substantially. Exact *P* values are provided in Supplementary Table S7. No multiple testing correction was applied.

**Fig. 5:**
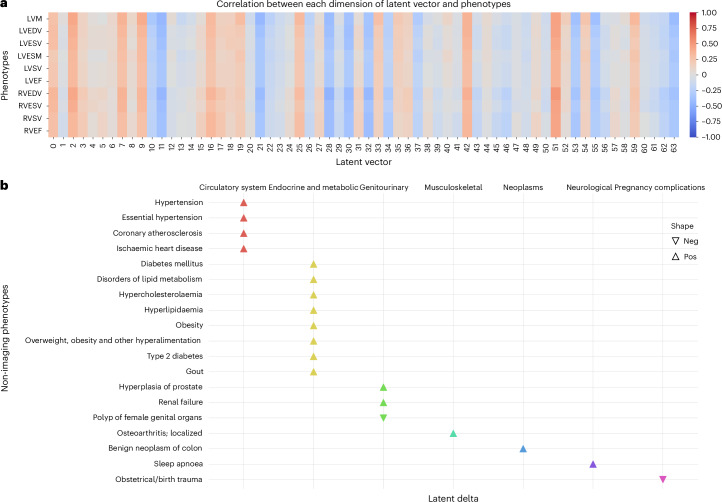
Association studies for the latent vector and the latent delta with imaging-derived phenotypes and clinical features. **a**, A heatmap of the Pearson correlation coefficients between imaging phenotypes and the 64-dimensional latent vector. The intensity of the colour reflects the magnitude and direction of the correlation, where blue signifies negative (Neg) correlations and red signifies positive (Pos) correlations. Darker shades indicate a stronger correlation between the vector and the phenotypes. **b**, PheWAS between the latent delta Δ*z* and unbiased categories of clinical features. The *y* axis lists the clinical outcomes where a signficiant association was identified. The *x* axis uses different colours to represent different disease categories. Each triangle denotes a notable PheWAS association, adjusted for multiple comparisons using the Bonferroni correction for 1,163 clinical features analysed. These clinical features encompass both clinical outcomes (for example, diseases and diagnoses) and phenotypes, covering a wide range of characteristics and measurements. The shape of each triangle indicates the direction of the effect. This analysis included 17,000 participants.

After demonstrating the generative capability of MeshHeart, we explore its potential for clinical applications, in particular using its latent space, which provides a low-dimensional representation of cardiac shape and motion. The latent feature analyses were conducted on 17,309 participants. More than half (58.5%) had a reported diagnosis of at least one disease. We use the latent vector *z* of each mesh sequence, a 64-dimensional vector, as the feature for correlation analysis and for cardiovascular disease classification. Figure 5a shows that the latent vector exhibits strong correlations with conventional imaging phenotypes, such as LVM, LVEDV and RVEDV. Figure 4 and Supplementary Table 7 compare the classification performance of six cardiac diseases when using different feature sets. The three evaluated feature sets include ‘phenotypes + confounders (age, sex, weight, height)’, ‘latent vector + confounders’ and ‘phenotypes + latent vector + confounders’. The classification performance is evaluated using the area under the curve (AUC) scores for three different classifiers: AdaBoost, linear discriminant analysis (LDA) and support vector machine (SVM). The six cardiovascular diseases include myocardial infarction (ICD-10 code I21), ischaemic heart diseases (I24), paroxysmal tachycardia (I47), atrial fibrillation and flutter (I48), hypertension (I10) and cardiac disease (I51). Figure 4 shows that using imaging phenotypes alone led to moderate AUC scores (for example, 0.8361 and 0.8201 for myocardial infarction and ischaemic heart diseases using with AdaBoost). Using the latent vector resulted in increased AUC scores (0.8557 and 0.8453). Combining both imaging phenotypes and the latent vector further improved the AUC scores (0.8762 and 0.8472), indicating the usefulness of the latent vector for cardiovascular disease classification. These results demonstrate the model’s ability to discriminate not only between normal and abnormal cardiac states but also among specific disease conditions.

For the AdaBoost classifier, using feature sets comprising the latent vector, as well as the combination of phenotypes and the latent vector, consistently outperformed the performance of the phenotypes set alone (for example, 0.8291 and 0.8316 for cardiac disease using latent vector and combined feature sets), implying that incorporating the latent vector improved the classification accuracy. The trend was particularly noticeable for myocardial infarction, hypertension and cardiac diseases, where the combined phenotypes and latent vector feature set substantially improved the AUC scores (0.8762, 0.7738 and 0.8316 for myocardial infarction, hypertension and cardiac disease). While the model was trained using a normal healthy heart dataset, it has learned a rich latent representation to encode diverse shape and motion patterns for different subpopulations in this large dataset. The resulting latent vector captures deviations in the latent space that are indicative of specific disease outcomes, as demonstrated by the experimental results. The LDA and SVM classifiers demonstrated that, among the three feature sets, the combined phenotypes and latent vector feature set achieved the highest AUC scores (for example, 0.6728 and 0.6479 for hypertension with LDA and SVM). However, for certain diseases such as ischaemic heart disease, classifiers using only phenotypes (for example, 0.7381 and 0.7123 for ischaemic heart diseases with LDA and SVM) outperformed those that used only the latent vector (0.7277 and 0.6975) but still fell short of their combination (0.7492 and 0.7214). Overall, the results show that, integrating imaging phenotypes, the latent vector along with confounders provides the best discriminative feature set for classification.

### Latent delta for PheWAS

For each individual heart, we use MeshHeart to generate a normal synthetic heart using the same clinical factors as this individual. This synthetic heart can be regarded as a personalized normative model learned from a specific subpopulation. We define the latent delta Δ*z* to be the difference between the latent vectors of an individual heart and its personalized norm, quantified using the Euclidean distance. The latent delta characterizes the deviation of the shape and motion patterns of an individual heart from the normal pattern for a subpopulation with the same clinical factors (Fig. 1c). A PheWAS was performed to explore the clinical relevance of Δ*z*, as shown in Fig. 5b. The PheWAS revealed notable associations between the latent delta Δ*z* and an unbiased set of clinical outcomes, including circulatory system diseases, endocrine and metabolic diseases, genitourinary diseases, musculoskeletal diseases and neoplasms.

The latent delta has been shown to correlate with phenotypes such as LVM and LVEF (Fig. 5a), which serve as indicators of cardiac structure and function. Conditions such as hypertension, lipid and cholesterol abnormalities and diabetes can induce changes in these cardiac phenotypes. For example, hypertension probably results in an increased LVM and may be linked to a reduced LVEF due to the heart’s adaptation to prolonged high blood pressure. In a similar vein, diabetes can exert metabolic stress on the heart, which can lead to changes in cardiac volume and ejection fraction. These modifications in the structure and motion patterns of the heart, as captured by the latent delta, provide a mechanistic explanation for the associations observed in the PheWAS results.

In Fig. 5b, the direction of effect shows the relationship between Δ*z* and the clinical outcome. A positive effect indicates that an increase in Δ*z* is associated with a higher probability of the outcome. By contrast, a negative effect indicates that a higher Δ*z* reduces the likelihood of the outcome. For example, a negative effect for birth trauma suggests that a higher Δ*z* is associated with a reduced likelihood of birth trauma. These directional effects provide insight into how deviations in cardiac structure and function relate to specific clinical outcomes, highlighting potential associations for further in-depth clinical investigation.

## Discussion

This work contributes to the growing field of generative artificial intelligence for science, with a specific application in cardiac imaging. The proposed MeshHeart model is a generative model that can facilitate improved understanding of the complexities of 3D + t cardiac shape and motion. In this study, we made four major contributions. First, we developed MeshHeart using a dataset of 38,309 participants from a large UK population^46^, capturing the variation in cardiac structures and clinical characteristics. Second, we demonstrated MeshHeart’s capability to generate a normal heart, accounting for clinical factors such as age, sex, weight and height. This established a personalized normative model for cardiac anatomy. Third, we investigated the latent vector of MeshHeart and demonstrated its associations with conventional imaging phenotypes and usefulness for enhancing disease classification performance. Finally, we propose a latent delta (Δ*z*) metric. This metric provides a way for quantifying the difference between an individual heart and the normative model, as well as for investigating the associations between the spatial–temporal characteristics of the heart and various health outcomes.

MeshHeart’s reconstruction capability was assessed using HD and ASSD metrics. Using these two metrics, we compared the model with other models along with an ablation study. Using geometric convolutions and a temporal transformer, the model reconstructed more accurate cardiac mesh sequences compared with other state-of-the-art models. This is is due to the reason that geometric convolutions are proficient in encoding mesh geometry, and the transformer is effective in capturing long-range temporal dependencies. The ablation study confirms the essential role of geometric convolutions and the temporal transformer in increasing the performance of the model, as detailed in Supplementary Table 6. We also compared MeshHeart against a previous work CHeart^42^. CHeart uses segmentation as a representation method for the cardiac structure, whereas MeshHeart uses the mesh representation. The results show that mesh provides a powerful representation for modelling the 3D geometry as well for tracking temporal motion, as it essentially allows the movement of each individual point to be monitored over time.

The generative capabilities of MeshHeart, as illustrated by the results in Fig. 3 and Supplementary Tables 3 and 4, demonstrate its proficiency as a generative model, able to replicate a normal heart on the basis of certain clinical factors including demographics (age and sex) and anthropometrics (weight and height). These four factors have shown strong correlations with heart structure and function across various individuals^9,52,53^. They form a reliable basis for constructing a normal heart model for an individual, as shown in Fig. 3b. Our analysis in Fig. 3a and Supplementary Tables 3 and 4 focused on age and sex, using WD and KL divergence to assess the similarity between the real and synthetic data distributions. Lower WD and KL metrics suggest that MeshHeart effectively represents demographic diversity, making the synthetic data beneficial for potential clinical and research purposes. The incorporation of additional clinical variables in the future, such as blood pressure and medical history, could improve the representation of cardiac health and diseases, thus enabling more potential applications for downstream tasks.

The latent vector obtained from the MeshHeart demonstrated its discriminative power for disease classification tasks. Incorporating the latent vector as feature substantially improves the classification accuracy for a range of cardiovascular conditions, as illustrated in Fig. 4. Although conventional imaging phenotypes can also be used as a feature set for the classification model, their classification performance was surpassed by the augmented feature set that also includes the latent vector, suggesting that the latent vector may contain some information not provided by the imaging phenotypes. Combining imaging phenotypes with the latent vector and confounders consistently achieved the best classification performance, regardless of the classification model used, demonstrating the benefit of integrating multiple data sources to represent the status of the heart. Some dimensions of the latent vector exhibit high correlations with conventional cardiac phenotypes, which are essential for assessing cardiovascular disease risk. The high correlation with the latent vector underscores their clinical analysis potential.

PheWAS uses a data-driven approach to uncover unbiased associations between cardiac deviations and disease diagnoses. Our analysis found that greater deviations in heart function are linked to increased risks of endocrine, metabolic and circulatory diseases. These cardiac diseases suggest underlying metabolic problems such as insulin resistance or metabolic disturbances observed in diabetes and obesity, which affect the structure and performance of the heart^54,55^. Likewise, they indicate wider circulatory conditions such as hypertension and atherosclerosis, which can lead to heart failure and ischaemic heart disease^56^. Understanding these relationships is crucial for risk stratification, personalized medicine and prevention strategies, highlighting the need for thorough cardiac evaluations in clinical management^57^.

Although this work advances the science in personalized cardiac modelling, there are several limitations. First, the personalized normative model relies on a restricted range of generating factors, including age, sex, weight and height, as we aim to develop a standard healthy heart. Including additional elements in the future such as diseases or environmental factors such as air pollution and noise^58^ could improve our understanding of their impacts on cardiac anatomy and function. Second, the model uses a cross-sectional dataset from the UK Biobank for both training and testing purposes. However, it does not include a benchmark for the progression of cardiac ageing, which could be addressed by using a longitudinal dataset to evaluate the model. Repeated scans are expected in the near future from the UK Biobank. Third, this study focuses on modelling the dynamic mesh sequence to describe cardiac shape and motion. It does not aim to model the underlying electrophysiology or biomechanics of the heart, which are also essential for cardiac modelling and understanding cardiac function^59,60^. In addition, the explainability of latent vectors could be explored, as understanding the specific information each latent dimension captures is crucial for clinical interpretation and validation. Finally, our method does not incorporate long-axis images, which limits its ability to capture the mitral, tricuspid or aortic valves for assessing valvular function. Mauger et al.^61^ used two-chamber and four-chamber long-axis images to identify tricuspid and mitral valve points, so that the motion of the valve points can be tracked and modelled using principal component analysis.

In conclusion, this study presents MeshHeart, a generative model for cardiac shape modelling. By training and evaluating the model on a population-level dataset from the UK Biobank, we demonstrate that MeshHeart not only achieves a high reconstruction accuracy but also excels in generating synthetic cardiac mesh sequences that closely resemble the real heart. The latent vector of the generative model and the latent delta metric provide new avenues of research to improve disease classification and personalized healthcare. These findings pave the way for future research on cardiac modelling and may inspire the development of generative modelling techniques for other types of biomedical data.

## Methods

### Generative model architecture

Figure 1a illustrates the architecture of the proposed generative model, MeshHeart. Given a set of clinical conditions *c*, our goal is to develop a model that can generate a dynamic 3D cardiac mesh sequence, *X*_0:*T*−1_ = {*x*_0_, *x*_1_, ⋯, *x*_*T*−1_}, where *T* denotes the number of time frames, that corresponds to the conditions *c*. Figure 1b shows an example of the input conditions and the generated mesh sequence. Without losing generality, we take age, sex, weight and height as conditions *c* in this work. Age, weight and height are continuous variables, whereas sex is a binary variable. Each cardiac mesh *x*_*t*_ = (*v*_*t*_, *e*_*t*_) is a graph with a set of vertices *v* and a set of edges *e* connecting them.

The proposed generative model consists of a mesh encoder *M*_enc_, a transformer encoder *T*_enc_, a condition encoder *C*_enc_, a transformer decoder *T*_dec_ and a mesh decoder *M*_dec_. These components are designed to work together to learn the probability distribution *p*_*θ*_(*x*∣*z*_*c*_) of the cardiac mesh sequence conditioned on clinical attributes, where *θ* represents the decoder parameters and *z*_*c*_ denotes the condition latent vector. The condition encoder *C*_enc_, implemented as a multilayer perceptron (MLP), maps the clinical conditions *c* into a condition latent vector *z*_*c*_.

The mesh encoder *M*_enc_, implemented as a GCN, processes the input cardiac mesh sequence *x*_0:*T*−1_. It extracts latent representations *z*_0:*T*−1_, where each vector *z*_*t*_ corresponds to a latent representation of the cardiac mesh at time frame *t*. These latent vectors serve as intermediate representations of the cardiac mesh sequence.

The latent vectors *z*_0:*T*−1_ from the mesh encoder are concatenated with the condition latent vector *z*_*c*_ to form a sequence of input tokens to the transformer encoder *T*_enc_. The transformer encoder *T*_enc_ captures temporal dependencies across the sequence, which comprises *L* layers of alternating blocks of multihead self-attention (MSA) and MLP. To ensure stability and effective learning, LayerNorm (LN) is applied before each block and residual connections are applied after each block. Similar to the class token in the vision transformer^62^, we append the input tokens *z*_0:*T*−1_ with two learnable parameters *μ*_token_ and *Σ*_token_, named distribution parameter tokens, which parameterize a Gaussian distribution over the latent space. In the transformer output layer, we extract the outputs from the distribution parameter tokens as distribution parameters *μ* and *Σ*. We then use the reparameterization trick^63^ to derive the latent *z*_*a*_ from *μ* and *Σ*, as shown in Fig. 1a. The encoding process is formulated as

1 $$\begin{array}{rcl}{z}_{{\mathrm{input}}}&=&[{\mu }_{{\mathrm{token}}};{\Sigma }_{{\mathrm{token}}};{z}_{0};{z}_{1};\ldots ;{z}_{T-1}]\\ {z}^{{\prime} l}&=&{\mathrm{MSA}}\left({\mathrm{LN}}\left({z}^{l-1}\right)\right)+{z}^{l-1},l=1,\ldots ,L\\ {z}^{l}&=&{\mathrm{LN}}\left[{\mathrm{MLP}}\left.\right({\mathrm{LN}}\left({z}^{{\prime} l}\right)\right]\\ {z}_{a}&=&\mu +\epsilon\Sigma ,\epsilon \sim {\mathcal{N}}(0,{\bf{1}})\end{array}.$$

where ~ means distributed as, indicating that the random variable *ε* follows a normal distribution, where the bold **1** denotes the identity matrix. The resulting latent vector *z*_*a*_, derived after the reparameterization step, captures the information about the distribution of the mesh sequence. This vector is concatenated with the condition latent vector *z*_*c*_ to form the input to the transformer decoder *T*_dec_. The decoder uses these concatenated vectors as keys and values in the self-attention layer, while sinusoidal temporal positional encodings^62^ serve as queries to incorporate temporal information. The temporal positional encoding *p*_*t*_ at time frame *t* is defined using the sinusoidal function with the same dimension *d* as *z*_*a*_:

2 $${{p}_{t}}^{(i)}=\left\{\begin{array}{ll}\sin \left(t/\text{10,000}^{2i/d}\right),\quad &\,\text{if}\,\,i=2k\\ \cos \left(t/\text{10,000}^{2i/d}\right),\quad &\,\text{if}\,\,i=2k+1\end{array}\right.,$$

where *i* denotes the dimension index. The transformer decoder outputs a sequence of latent vectors, each corresponding to a mesh representation at a timepoint of the cardiac cycle. The latent vectors generated by the transformer decoder are passed through the mesh decoder *M*_dec_, composed of fully connected (FC) layers, to reconstruct the 3D + t cardiac mesh sequence ${X}_{0:T-1}^{{\prime} }$.

### Probabilistic modelling and optimization

Following the VAE formulation^63,64^, we assume a prior distribution *p*(*z*_*a*_) over the latent variable *z*_*a*_. The prior *p*(*z*_*a*_), together with the decoder (constructed by *T*_dec_ and *M*_dec_), defines the joint distribution *p*(*x*, *z*_*a*_∣*z*_*c*_). To train the model and perform inference, we need to compute the posterior distribution *p*(*z*_*a*_∣*x*, *z*_*c*_), which is generally intractable. To turn the intractable posterior inference problem *p*(*z*_*a*_∣*x*, *z*_*c*_) into a tractable problem, we introduce a parametric encoder model (constructed by *C*_enc_, *M*_enc_ and *T*_enc_) *q*_*ϕ*_(*z*_*a*_∣*x*, *z*_*c*_) with *ϕ* to be the variational parameters, which approximates the true but intractable posterior distribution *p*(*z*_*a*_∣*x*, *z*_*c*_) of the generative model, given an input *x* and conditions *c*:

3 $${q}_{\phi }({z}_{a}| x,{z}_{c})\approx {p}_{\theta }({z}_{a}| x,{z}_{c}),$$

where *q*_*ϕ*_(*z*_*a*_∣*x*, *z*_*c*_) often adopts a simpler form, for example the Gaussian distribution^63,64^. By introducing the approximate posterior *q*_*ϕ*_(*z*_*a*_∣*x*, *z*_*c*_), the log-likelihood of the conditional distribution *p*_*θ*_(*x*∣*z*_*c*_) for input data *x*, also known as evidence, can be formulated as

4 $$\begin{array}{rcl}\log {p}_{\theta }(x| {z}_{c})&=&{{\mathbb{E}}}_{{z}_{a} \sim {q}_{\phi }({z}_{a}| x,{z}_{c})}\log \left[{p}_{\theta }(x| {z}_{c})\right]\\ &=&{{\mathbb{E}}}_{{z}_{a} \sim {q}_{\phi }({z}_{a}| x,{z}_{c})}\log \left[\frac{{p}_{\theta }(x,{z}_{a}| {z}_{c})}{{q}_{\phi }({z}_{a}| x,{z}_{c})}\right]+{{\mathbb{E}}}_{{z}_{a} \sim {q}_{\phi }({z}_{a}| x,{z}_{c})}\log \left[\frac{{q}_{\phi }({z}_{a}| x,{z}_{c})}{{p}_{\theta }({z}_{a}| x,{z}_{c})}\right]\end{array},$$

where the second term denotes the KL divergence *D*_KL_(*q*_*ϕ*_∥*p*_*θ*_) between *q*_*ϕ*_(*z*_*a*_∣*x*, *z*_*c*_) and *p*_*θ*_(*z*_*a*_∣*x*, *z*_*c*_)^63,64^. It is non-negative and zero only if the approximate posterior *q*_*ϕ*_(*z*_*a*_∣*x*, *z*_*c*_) equals the true posterior distribution *p*_*θ*_(*z*_*a*_∣*x*, *z*_*c*_). Due to the non-negativity of the KL divergence, the first term in equation (4) is the lower bound of the evidence $\log [{p}_{\theta }(x| {z}_{c})]$, known as the evidence lower bound (ELBO). Instead of optimizing the evidence $\log [{p}_{\theta }(x| {z}_{c})]$, which is often intractable, we optimize the ELBO as follows:

5 $$\mathop{\min }\limits_{\theta ,\phi }{\mathrm{ELBO}}=-\log [{p}_{\theta }(x| {z}_{c})]+{D}_{{\mathrm{KL}}}.$$

### Training loss function

Based on the ELBO, we define the concrete training loss function, which combines the mesh reconstruction loss ${{\mathcal{L}}}_{{\mathrm{R}}}$, the KL loss ${{\mathcal{L}}}_{{\mathrm{KL}}}$ and a mesh smoothing loss ${{\mathcal{L}}}_{{\mathrm{S}}}$. The mesh reconstruction loss ${{\mathcal{L}}}_{{\mathrm{R}}}$ is defined as the Chamfer distance between the reconstructed mesh sequence ${X}_{0:T-1}^{{\prime} }=({V}^{{\prime} },{E}^{{\prime} })$ and the ground truth *X*_0:*T*−1_ = (*V*, *E*), formulated as ${{\mathcal{L}}}_{{\mathrm{R}}}=\frac{1}{T}\mathop{\sum }\nolimits_{t = 0}^{T-1}{D}_{{\mathrm{cham}}}({V}_{t}^{{\prime} },{V}_{t})$, where *D*_cham_ denotes the Chamber distance^65^, ${V}_{t}^{{\prime} }$ and *V*_*t*_ denote the mesh vertex coordinates for the reconstruction and the ground truth, respectively:

6 $${D}_{{\mathrm{cham}}}({V}_{t},{V}_{t}^{{\prime} })=\frac{1}{\left\vert {V}_{t}\right\vert }\sum _{{v}_{t}\in {V}_{t}}\mathop{\min }\limits_{{v}_{t}^{{\prime} }\in {V}_{t}^{{\prime} }}{\left\Vert {v}_{t}-{v}_{t}^{{\prime} }\right\Vert }_{2}+\frac{1}{\left\vert {V}_{t}^{{\prime} }\right\vert }\sum _{{v}_{t}^{{\prime} }\in {V}_{t}^{{\prime} }}\mathop{\min }\limits_{{v}_{t}\in {V}_{t}}{\left\Vert {v}_{t}^{{\prime} }-{v}_{t}\right\Vert }_{2}.$$

In the VAE, the distribution of the latent space for *z*_*a*_ is encouraged to be close to a prior Gaussian distribution. The KL divergence is defined between the latent distribution and the Gaussian prior distribution. To control the trade-off between distribution fitting and diversity, we adopt the *β*-VAE formulation^64^. The KL loss ${{\mathcal{L}}}_{{\mathrm{KL}}}$ is formulated as

7 $${{\mathcal{L}}}_{{\mathrm{KL}}}=\beta \cdot {\mathrm{KL}}({\mathcal{N}}(\;\mu ,\Sigma )\parallel {\mathcal{N}}(0,{\bf{1}})),$$

which encourages the latent space ${\mathcal{N}}(\;\mu ,\Sigma )$ to be close to the prior Gaussian distribution ${\mathcal{N}}(0,{\bf{I}})$.

The Laplacian smoothing loss penalizes the difference between neighbouring vertices such as sharp changes on the mesh^66,67^. It is defined as

8 $$\begin{array}{rcl}{{\mathcal{L}}}_{{\mathrm{S}}}&=&\frac{1}{T}\mathop{\sum }\limits_{t=0}^{T-1}{D}_{{\mathrm{smooth}}}({V}_{t}^{{\prime} },{E}_{t}^{{\prime} })\\ {D}_{{\mathrm{smooth}}}(V,E)&=&\mathop{\sum}\limits _{{v}_{i}\in V}\frac{1}{| V| }{\left\Vert\mathop{\sum}\limits_{j\in {N}_{i}}\frac{1}{| {N}_{i}| }({v}_{j}-{v}_{i})\right\Vert }_{2}\end{array},$$

where *N*_*i*_ denotes the neighbouring vertices adjacent to *v*_*i*_. The total loss function *L* is a weighted sum of the three loss terms

9 $${\mathcal{L}}={{\mathcal{L}}}_{{\mathrm{R}}}+{{\mathcal{L}}}_{{\mathrm{KL}}}+{\lambda }_{{\mathrm{s}}}\cdot {{\mathcal{L}}}_{{\mathrm{S}}}.$$

In terms of implementation, the mesh encoder *M*_enc_ has three GCN layers and one FC layer. The mesh decoder *M*_dec_ is composed of five FC layers. The transformer encoder *T*_enc_ and decoder *T*_dec_ consist of two layers, four attention heads, a feed-forward size of 1,024 and a dropout rate of 0.1. The latent vector dimensions for the mesh and condition were set to 64 and 32, respectively. The model contains approximately 69.71 million parameters and was trained on an NVIDIA RTX A6000 graphics processing unit (48 GB) using the Adam optimizer with a fixed learning rate of 10^−4^ for 300 epochs. Training was performed with a batch size of one cardiac mesh sequence, consisting of 50 time frames. The cardiac mesh at each time frame consists of 22,043 vertices and 43,840 faces. The weights *β* and *λ*_s_ in the loss function were empirically set to 0.01 and 1.

### Personalized normative model, latent vector and delta

MeshHeart is trained on a large population of asymptomatic hearts. Once trained, it can be used as a personalized normative model to generate a synthetic mesh sequence of a normal heart with certain attributes *c*, including age, sex, weight and height. For each real heart, we can then compare the real cardiac mesh sequence with the synthetic normal mesh sequence of the same attributes, to understand the deviation of the real heart from its personalized normative pattern.

To represent a cardiac mesh sequence in a low-dimensional latent space, we extract a latent vector after the transformer encoder *T*_enc_ but before the reparameterization step. The latent vector is calculated as the mean of the latent vectors at the transformer encoder output layer across 50 time frames. For calculating the latent delta, we quantify the deviation of the latent vector of the real heart to the latent vector of a group of synthetic hearts of the same attributes. Given conditions *c*, 100 samples of the latent variable *z*_*a*_ are drawn from a standard Gaussian distribution, ${z}_{a} \sim {\mathcal{N}}({\bf{0}},{\bf{I}})$, where *z*_*a*_ denotes the latent space after reparameterization in the VAE formulation. Each sample *z*_*a*_ is concatenated with the condition latent vector *z*_*c*_ and passed through the transformer decoder and mesh decoder to generate a synthetic cardiac mesh sequence. After synthetic mesh generation, each synthetic mesh sequence is provided to the mesh encoder *M*_enc_ and transformer encoder *T*_enc_, to generate latent vectors across 50 time frames at the transformer output later, subsequently averaged to form the latent vector *z*^synth^. The real heart mesh sequence is provided to the mesh encoder *M*_enc_ and transformer encoder *T*_enc_ for calculating the latent vector *z*^real^ in the same manner.

With the latent vector *z*^real^ for the real heart and the latent vector *z*^synth^ for the synthetic heart, we define the latent vector as the Euclidean distance between *z*^real^ and *z*^synth^. As we draw 100 synthetic samples to represent a subpopulation with the same attributes, the latent delta Δ*z* is defined as

10 $$\Delta z={\left\Vert {z}^{{\rm{real}}}-\frac{1}{100}\mathop{\sum }\limits_{i = 1}^{100}{z}_{i}^{{\rm{synth}}}\right\Vert }_{2},$$

where *i* denotes the sample index. The latent delta Δ*z* provides a robust metric to evaluate individual differences in cardiac structure and motion, quantifying the deviation of the real heart from its personalized normative model.

### Data and experiments

This study used a dataset of 38,309 participants obtained from the UK Biobank^46^. Each participant underwent cine cardiac MR (CMR) imaging scans. From the cine CMR images, a 3D mesh sequence is derived to describe the shape and motion of the heart. The mesh sequence covers three anatomical structures, LV, Myo and RV. Each sequence contains 50 time frames over the course of a cardiac cycle. To derive cardiac meshes from the CMR images, automated segmentation^68^ was applied to the images. The resulting segmentations were enhanced using an atlas-base approach^69^, by registering multiple high-resolution cardiac atlases^69,70^ onto the segmentations followed by label fusion, resulting in high-resolution segmentations. A 3D template mesh^70^ was then fitted to the high-resolution segmentations at the ED and ES frames using non-rigid image registration, generating ED and ES cardiac meshes. Subsequently, motion tracking was performed using Deepali^71^, a graphics-processing-unit-accelerated version of the non-rigid registration toolbox MIRTK^72^, on cardiac segmentations across the time frames. Deformation fields were derived using a free-form deformation model with a control point spacing of [8, 8, 8]. The registration objective function included Dice similarity as the primary similarity metric and B-spline bending energy regularization with a weight of 0.01. The deformation fields were derived between time frames and applied to propagate the ED mesh and ES mesh across the cardiac cycle. The proposed meshes were averaged using weighted interpolation based on temporal proximity to ED and ES^9^ to ensure temporal smoothness of the resulting mesh sequence. All cardiac meshes maintained the same geometric structure.

The dataset was partitioned into training, validation and test sets for developing the MeshHeart model and a clinical analysis set for evaluating its performance for disease classification task. In brief, MeshHeart was trained on 15,000 healthy participants from the Cheadle imaging centre. For parameter tuning and performance evaluation, MeshHeart was evaluated on a validation set of 2,000 and a test set of 4,000 healthy participants, from three different sites, Cheadle, Reading and Newcastle centres. For clinical analysis, including performing the disease classification study and latent delta PheWAS, we used a separate set of 17,309 participants from the three imaging centres, including 7,178 healthy participants and 10,131 participants with cardiac diseases and hypertension. PheWAS was undertaken using the PheWAS R package with clinical outcomes and coded phenotypes converted to 1,163 categorical PheCodes. *P* values were deemed significant with Bonferroni adjustment for the number of PheCodes. The details of the dataset split and the definition of disease code are described in Supplementary Table 1.

### Method comparison

To compare the generation performance of MeshHeart, we adapt three state-of-the-art generative models originally proposed for other tasks: (1) Action2Motion^47^, originally developed for human motion generation; (2) ACTOR^27^, developed for human pose and motion generation; and (3) CHeart^42^, developed for the generation of cardiac segmentation maps, instead of cardiac meshes. We modified these models to adapt to the cardiac mesh generation task.

## Supplementary information

Supplementary Information Supplementary Tables 1–8 and Figs. 1–4.

## Data Availability

The raw imaging data and non-imaging participant characteristics are available from UK Biobank to approved researchers via a standard application process at http://www.ukbiobank.ac.uk/register-apply.

## Code availability

The code for this research is available via GitHub at https://github.com/MengyunQ/MeshHeart and via Zenodo at 10.5281/zenodo.15122485 (ref. ^73^).
