# Supplementary material for: A personalized time-resolved 3D mesh generative model for unveiling normal heart dynamics
Source: Nat Mach Intell. 2025 May 19;7(5):800–11. doi: 10.1038/s42256-025-01035-5 (PMC12101970; doi:10.1038/s42256-025-01035-5)
Supplement: Supplementary file 1 — Supplementary Tables 1–8 and Figs. 1–4. [file 42256_2025_1035_MOESM1_ESM.pdf]

nature machine intelligence

Supplementary information https://doi.org/10.1038/s42256-025-01035-5

A personalized time-resolved 3D mesh generative model for unveiling normal heart dynamics

In the format provided by the authors and unedited

A Personalised 3D+t Mesh Generative Model for Unveiling Normal Heart Dynamics: Supplemental Document

1. DATASET

Table S1. Summary information on the participants from the UK Biobank involved in the study. It encompasses a total of 38,309 participants, divided into groups for training, validation, testing, and clinical analysis. Healthy participants were defined as those without documented cardiovascular conditions, based on ICD-10 codes recorded in the UK Biobank fields "Diagnoses - main ICD10" and "Diagnoses - secondary ICD10." Specifically, we excluded individuals with codes for chronic rheumatic heart diseases (I05–I09), hypertensive diseases (I10–I15), ischaemic heart diseases (I20–I25), pulmonary heart disease and diseases of pulmonary circulation (I26–I28), and other forms of heart disease (I30–I52).

Participants in the training, validation, and testing groups are all healthy, while the clinical analysis group includes both healthy individuals and those with illnesses. The table also outlines the distribution of participants by sex and by assessment centre location (Cheadle, Reading, Newcastle).

	Total number	Sex		Age (years)		Weight (kg)		Height (cm)	
Dataset	38,309	Female	Male	Mean	STD	Mean	STD	Mean	STD
Train	15,000	8,296	6,704	62.58	7.53	74.71	14.61	168.99	9.22
Validation	2,000	1,147	853	63.95	7.65	73.70	14.26	168.56	9.23
Test	4,000	2,231	1,769	63.94	7.53	74.00	14.23	168.83	9.26
Clinical analysis	17,309	8,237	9,072	65.46	7.30	78.17	15.72	169.45	9.33

Health condition							Centre		
Healthy	Cardiac diseases	Hypertension	Myocardial infarction	Ischemic heart diseases	Paroxysmal tachycardia	Atrial fibrillation and flutter	Cheadle	Reading	Newcastle
15,000	0	0	0	0	0	0	15,000	0	0
2,000	0	0	0	0	0	0	439	982	1,058
4,000	0	0	0	0	0	0	914	982	2,104
7,178	2,463	8,300	613	1,688	219	1,034	8,469	2,783	6,057

2. ARCHITECTURE

Table S2. Architecture comparison of Action2Motion, ACTOR, and MeshHeart.

Model	Spatial Encoder	Temporal Encoder
Action2Motion	Linear fully connected layers	GRU
Actor	Linear fully connected layers	Transformer
MeshHeart	Graph Convolutional Network (GCN)	Transformer

3. RESULTS

Table S3. Comparison of cardiac mesh reconstruction accuracy. The Hausdorff distance (HD) and average symmetric surface distance (ASSD) are reported for the whole sequence and at the end-diastolic frame (ED) and the end-systolic frame (ES) frames. The mean value and standard deviation are reported. For all metrics, lower mean values indicate better results.

Methods	HD (unit: mm)↓				ASSD (unit: mm)↓			
	Average	LV	Myo	RV	Average	LV	Myo	RV
Mean across sequence								
Action2Motion	$4.389_{\pm 0.946}$	$\mathbf{4.956}_{\pm \mathbf{1.647}}$	$4.182_{\pm 1.110}$	$5.444_{\pm 1.449}$	$2.043_{\pm 0.419}$	$2.358_{\pm 0.701}$	$2.004_{\pm 0.476}$	$2.430_{\pm 0.563}$
ACTOR	$4.365_{\pm 1.046}$	$5.454_{\pm 1.929}$	$4.276_{\pm 1.101}$	$5.029_{\pm 1.316}$	$2.029_{\pm 0.451}$	$2.438_{\pm 0.732}$	$2.012_{\pm 0.476}$	$2.371_{\pm 0.635}$
CHeart	$6.502_{\pm 2.114}$	$7.217_{\pm 2.797}$	$6.828_{\pm 2.675}$	$7.360_{\pm 2.395}$	$2.821_{\pm 0.791}$	$3.622_{\pm 1.597}$	$2.948_{\pm 1.010}$	$3.338_{\pm 1.003}$
Proposed	$\mathbf{4.163}_{\pm \mathbf{0.827}}$	$5.477_{\pm 1.802}$	$\mathbf{4.060}_{\pm \mathbf{0.883}}$	$\mathbf{4.762}_{\pm \mathbf{1.207}}$	$\mathbf{1.934}_{\pm \mathbf{0.359}}$	$\mathbf{2.350}_{\pm \mathbf{0.565}}$	$\mathbf{1.927}_{\pm \mathbf{0.386}}$	$\mathbf{2.257}_{\pm \mathbf{0.625}}$
ED frame								
Action2Motion	$4.456_{\pm 1.198}$	$6.077_{\pm 2.708}$	$4.266_{\pm 1.308}$	$5.324_{\pm 1.659}$	$1.964_{\pm 0.469}$	$2.491_{\pm 0.978}$	$1.891_{\pm 0.492}$	$2.439_{\pm 0.785}$
ACTOR	$4.267_{\pm 1.244}$	$\mathbf{5.784}_{\pm \mathbf{2.194}}$	$4.155_{\pm 1.333}$	$\mathbf{4.989}_{\pm \mathbf{1.486}}$	$1.939_{\pm 0.470}$	$2.452_{\pm 0.762}$	$1.891_{\pm 0.488}$	$2.340_{\pm 0.690}$
CHeart	$5.255_{\pm 1.660}$	$6.041_{\pm 2.491}$	$5.629_{\pm 2.295}$	$6.215_{\pm 2.160}$	$2.296_{\pm 0.664}$	$3.011_{\pm 1.373}$	$2.436_{\pm 0.917}$	$2.721_{\pm 0.824}$
Proposed	$\mathbf{4.240}_{\pm \mathbf{0.815}}$	$5.798_{\pm 2.081}$	$\mathbf{4.063}_{\pm \mathbf{1.092}}$	$5.018_{\pm 0.994}$	$\mathbf{1.903}_{\pm \mathbf{0.311}}$	$\mathbf{2.395}_{\pm \mathbf{0.629}}$	$\mathbf{1.856}_{\pm \mathbf{0.424}}$	$\mathbf{2.317}_{\pm \mathbf{0.510}}$
ES frame								
Action2Motion	$4.780_{\pm 1.203}$	$5.664_{\pm 2.426}$	$4.480_{\pm 1.164}$	$5.964_{\pm 1.707}$	$2.220_{\pm 0.557}$	$2.446_{\pm 0.802}$	$2.110_{\pm 0.530}$	$2.765_{\pm 0.871}$
ACTOR	$4.416_{\pm 1.195}$	$5.249_{\pm 2.163}$	$4.383_{\pm 1.265}$	$5.003_{\pm 1.521}$	$2.084_{\pm 0.551}$	$2.448_{\pm 0.849}$	$2.107_{\pm 0.588}$	$2.355_{\pm 0.750}$
CHeart	$5.525_{\pm 1.410}$	$6.229_{\pm 2.348}$	$5.894_{\pm 1.916}$	$5.763_{\pm 1.353}$	$2.462_{\pm 0.553}$	$3.001_{\pm 1.399}$	$2.672_{\pm 0.807}$	$2.606_{\pm 0.548}$
Proposed	$\mathbf{4.174}_{\pm \mathbf{1.305}}$	$\mathbf{5.239}_{\pm \mathbf{2.058}}$	$\mathbf{4.148}_{\pm \mathbf{1.223}}$	$\mathbf{4.626}_{\pm \mathbf{1.675}}$	$\mathbf{1.932}_{\pm \mathbf{0.546}}$	$\mathbf{2.325}_{\pm \mathbf{0.644}}$	$\mathbf{1.964}_{\pm \mathbf{0.530}}$	$\mathbf{2.177}_{\pm \mathbf{0.811}}$

Table S4. Wasserstein distance (WD) and the Kullback-Leibler(KL) divergence between generated and real data distribution for phenotypes including LVEDV, LVESV, LVEF, LVM, RVEDV, RVESV and RVEF against age.

Phenotypes	LVEDV	LVESV	LVEF	LVM	RVEDV	RVESV	RVEF
WD							
Action2Motion	59.165(3.317)	24.112(1.942)	6.545(0.799)	38.999(1.942)	61.562(3.155)	32.287(2.439)	3.879(0.198)
ACTOR	51.865(3.195)	27.171(1.602)	7.318(0.836)	38.935(2.692)	55.637(3.163)	33.883(2.302)	4.644(0.195)
CHeart	74.253(15.226)	37.949(7.567)	6.769(0.613)	61.475(13.495)	83.315(19.221)	55.502(12.615)	7.229(0.712)
MeshHeart	50.898(5.245)	24.389(2.235)	5.706(0.898)	35.376(3.433)	49.000(4.495)	29.853(2.517)	3.704(0.157)
KL							
Action2Motion	0.034(0.004)	0.047(0.007)	0.037(0.003)	0.035(0.006)	0.036(0.006)	0.033(0.003)	0.009(0.001)
ACTOR	0.032(0.004)	0.046(0.007)	0.034(0.003)	0.035(0.006)	0.034(0.006)	0.031(0.003)	0.008(0.001)
CHeart	0.056(0.012)	0.057(0.010)	0.038(0.004)	0.042(0.007)	0.041(0.006)	0.038(0.004)	0.009(0.001)
MeshHeart	0.003(0.002)	0.005(0.003)	0.031(0.003)	0.004(0.002)	0.004(0.002)	0.006(0.004)	0.002(0.001)

Table S5. WD and KL divergence between generated and real data distribution for phenotypes including LVEDV, LVESV, LVEF, LVM, RVEDV, RVESV and RVEF against sex.

Phenotypes	LVEDV	LVESV	LVEF	LVM	RVEDV	RVESV	RVEF
				WD			
Action2Motion	69.308(13.708)	37.827(7.963)	6.002(0.547)	61.447(12.225)	4.100(0.604)	80.358(18.670)	54.744(12.425)
ACTOR	69.664(7.584)	38.046(4.284)	6.837(0.865)	61.163(7.737)	5.961(0.393)	83.675(10.133)	51.397(7.384)
CHeart	72.226(4.836)	36.226(4.012)	6.137(0.865)	59.374(6.425)	5.538(0.715)	83.089(7.143)	49.712(6.227)
Proposed	54.588(9.074)	29.876(4.814)	5.772(0.933)	46.694(8.943)	31.574(69.454)	63.719(11.153)	39.798(7.892)
				KL			
Action2Motion	0.029(0.003)	0.040(0.004)	0.037(0.003)	0.027(0.003)	0.028(0.003)	0.008(0.000)	0.030(0.002)
ACTOR	0.029(0.004)	0.041(0.005)	0.032(0.002)	0.027(0.003)	0.028(0.003)	0.009(0.000)	0.031(0.002)
CHeart	0.054(0.012)	0.055(0.007)	0.037(0.003)	0.037(0.004)	0.037(0.004)	0.010(0.000)	0.037(0.003)
Proposed	0.015(0.004)	0.021(0.006)	0.030(0.002)	0.015(0.005)	0.016(0.005)	0.017(0.006)	0.034(0.010)

Table S6. Ablation studies. We compare various architectural designs, such as the encoders, decoders and different components of the model. Each row shows the performance after changing one component, compared to the default proposed model.

Architecture	Mean across sequence		ED frame		ES frame	
	HD (mm)↓	ASSD (mm)↓	HD (mm)↓	ASSD (mm)↓	HD (mm)↓	ASSD (mm)↓
M_{enc}: Linear	$5.707_{\pm 1.234}$	$2.567_{\pm 0.548}$	$7.210_{\pm 2.375}$	$2.867_{\pm 1.001}$	$7.730_{\pm 2.164}$	$3.494_{\pm 0.933}$
M_{enc}: CNN	$5.268_{\pm 1.613}$	$2.345_{\pm 0.673}$	$6.906_{\pm 2.335}$	$2.910_{\pm 1.035}$	$5.293_{\pm 1.896}$	$2.435_{\pm 0.851}$
$T_{\text{enc,dec}}$: GRU	$4.720_{\pm 1.619}$	$2.161_{\pm 0.713}$	$4.436_{\pm 1.710}$	$1.974_{\pm 0.721}$	$4.759_{\pm 1.759}$	$2.263_{\pm 0.778}$
$T_{\text{enc,dec}}$: LSTM	$5.015_{\pm 1.919}$	$2.254_{\pm 0.834}$	$4.700_{\pm 1.792}$	$2.060_{\pm 0.767}$	$5.046_{\pm 2.028}$	$2.350_{\pm 0.898}$
Proposed	$\mathbf{4.163_{\pm 0.827}}$	$\mathbf{1.934_{\pm 0.359}}$	$4.240_{\pm 0.815}$	$1.903_{\pm 0.311}$	$\mathbf{4.174_{\pm 1.305}}$	$\mathbf{1.932_{\pm 0.546}}$
1) w/out$\mathcal{L}_S$	$4.779_{\pm 1.147}$	$2.174_{\pm 0.604}$	$4.493_{\pm 1.527}$	$1.985_{\pm 0.611}$	$4.809_{\pm 1.650}$	$2.260_{\pm 0.689}$
2) w/out $\mu_{\text{token}}, \Sigma_{\text{token}}$	$4.333_{\pm 0.966}$	$2.053_{\pm 0.411}$	$\mathbf{3.604_{\pm 0.975}}$	$\mathbf{1.740_{\pm 0.432}}$	$5.257_{\pm 1.305}$	$2.444_{\pm 0.502}$

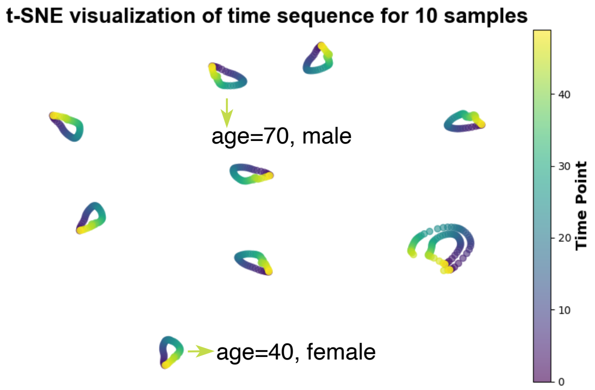

Fig. S1. t-SNE visualization of cardiac mesh sequences from 10 subjects generated randomly by MeshHeart. For each subject, a closed trajectory is shown, which is formed of latent vectors from the Transformer decoder across time frames. The latent vectors are mapped in a two-dimensional space by t-SNE.

Table S7. A comparison of the performance in disease classification, measured by AUC (area under the curve) scores, when various feature sets are utilized. The three feature sets considered are "Phenotypes + Confounders", "Latent vector + Confounders", and "Phenotypes + Latent vector + Confounders", tested with three different classifiers: AdaBoost, LDA, and SVM. The six cardiovascular diseases evaluated include myocardial infarction (ICD-10 code I21), ischemic heart diseases (I24), paroxysmal tachycardia (I47), atrial fibrillation and flutter (I48), hypertension (I10), and cardiac disease (I51). A single asterisk (*) indicates a statistically significant improvement ($p < 0.05$) over one other feature set, and a double asterisk (**) indicates a significant improvement over both, based on two-sided DeLong's test.

Classifier	Feature sets	Myocardial infarction	Ischemic heart diseases	Paroxysmal tachycardia	Atrial fibrillation and flutter	Hypertension	Cardiac disease
AdaBoost	Phenotypes + Confounders	0.8225	0.8083	0.7743	0.7983	0.7419	0.7786
	Latent vector + Confounders	0.8342	0.8122	0.7783	0.7971	0.7571	0.7803
	Phenotypes + Latent vector + Confounders	**0.8463***	**0.8083**	**0.8303****	**0.8145**	**0.7637***	**0.7985***
	p-value (vs baseline)	0.043	0.12	0.032	0.052	0.039	0.041
LDA	Phenotypes + Confounders	0.7265	0.7490	0.5805	0.7019	0.6937	0.7167
	Latent vector + Confounders	0.7172	0.7550	**0.6357***	0.7211	0.6998	0.7194
	Phenotypes + Latent vector + Confounders	**0.7296**	**0.7557**	0.6297*	**0.7304***	**0.7109**	**0.7379***
	p-value (vs baseline)	0.078	0.065	0.021	0.038	0.067	0.026
SVM	Phenotypes + Confounders	0.7023	0.7191	0.5837	0.7014	0.6893	0.7037
	Latent vector + Confounders	0.7086	0.7285	0.5818	0.7125	0.7013	0.6989
	Phenotypes + Latent vector + Confounders	**0.7172***	**0.7300**	**0.6351****	**0.7126**	**0.7020**	**0.7243**
	p-value (vs baseline)	0.046	0.058	0.006	0.062	0.053	0.051

Table S8. Comparison of cardiac mesh reconstruction accuracy on the UK Digital Heart dataset (315 subjects). The Hausdorff distance (HD) and average symmetric surface distance (ASSD) are reported for the whole sequence and at the end-diastolic frame (ED) and the end-systolic frame (ES). The mean value and standard deviation are reported. For all metrics, lower mean values indicate better results.

Methods	HD (unit: mm)↓				ASSD (unit: mm)↓			
	Avg	LV	Myo	RV	Avg	LV	Myo	RV
Mean across sequence								
ACTOR	$6.316_{\pm1.454}$	$8.150_{\pm1.548}$	$6.153_{\pm1.401}$	$7.510_{\pm2.160}$	$2.735_{\pm0.522}$	$3.591_{\pm0.723}$	$2.627_{\pm0.483}$	$3.526_{\pm1.028}$
Action2Motion	$5.504_{\pm1.264}$	$5.395_{\pm1.219}$	$5.236_{\pm1.370}$	$7.035_{\pm2.092}$	$2.454_{\pm0.477}$	$2.563_{\pm0.542}$	$2.338_{\pm0.460}$	$3.167_{\pm0.873}$
CHeart	$9.567_{\pm1.616}$	$12.113_{\pm2.051}$	$11.165_{\pm2.199}$	$13.405_{\pm2.525}$	$4.004_{\pm0.539}$	$6.219_{\pm1.159}$	$4.566_{\pm0.761}$	$5.467_{\pm1.040}$
MeshHeart	**$5.176_{\pm1.403}$**	**$5.360_{\pm1.443}$**	**$4.947_{\pm1.476}$**	**$6.428_{\pm2.438}$**	**$2.326_{\pm0.517}$**	**$2.522_{\pm0.579}$**	**$2.219_{\pm0.459}$**	**$2.977_{\pm1.050}$**
ED frame								
ACTOR	$6.024_{\pm1.773}$	$7.894_{\pm1.776}$	$5.663_{\pm1.794}$	$7.752_{\pm2.597}$	**$2.494_{\pm0.549}$**	$3.319_{\pm0.767}$	**$2.365_{\pm0.539}$**	**$3.344_{\pm1.012}$**
Action2Motion	$6.349_{\pm1.356}$	$7.684_{\pm1.808}$	$5.948_{\pm1.556}$	$7.741_{\pm2.356}$	$2.527_{\pm0.482}$	$3.341_{\pm0.907}$	$2.415_{\pm0.515}$	$3.369_{\pm0.882}$
CHeart	$9.927_{\pm1.813}$	$12.663_{\pm2.412}$	$11.825_{\pm2.460}$	$15.107_{\pm2.833}$	$4.160_{\pm0.711}$	$6.524_{\pm1.375}$	$4.956_{\pm1.084}$	$5.846_{\pm1.125}$
MeshHeart	**$5.829_{\pm1.416}$**	**$6.541_{\pm1.590}$**	**$5.568_{\pm1.547}$**	**$7.420_{\pm2.463}$**	$2.727_{\pm0.487}$	**$3.003_{\pm0.661}$**	$2.530_{\pm0.467}$	$3.800_{\pm1.066}$
ES frame								
ACTOR	$6.222_{\pm1.215}$	$8.635_{\pm2.032}$	$6.169_{\pm1.323}$	$7.410_{\pm1.967}$	$2.905_{\pm0.527}$	$3.857_{\pm0.853}$	$2.899_{\pm0.555}$	$3.528_{\pm0.957}$
Action2Motion	$5.439_{\pm1.288}$	$5.189_{\pm1.384}$	$5.153_{\pm1.286}$	$6.698_{\pm2.053}$	$2.504_{\pm0.540}$	**$2.402_{\pm0.619}$**	$2.432_{\pm0.546}$	$3.049_{\pm0.876}$
CHeart	$8.801_{\pm1.644}$	$10.848_{\pm2.111}$	$10.052_{\pm2.224}$	$11.439_{\pm2.358}$	$3.780_{\pm0.459}$	$5.600_{\pm1.185}$	$4.200_{\pm0.621}$	$4.837_{\pm0.909}$
MeshHeart	**$4.921_{\pm1.130}$**	**$5.103_{\pm1.502}$**	**$4.865_{\pm1.282}$**	**$5.939_{\pm2.109}$**	**$2.331_{\pm0.462}$**	$2.579_{\pm0.786}$	**$2.345_{\pm0.499}$**	**$2.729_{\pm0.860}$**

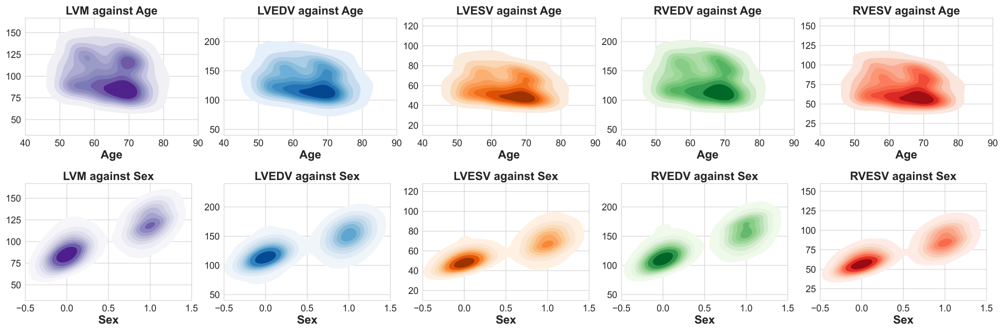

Fig. S2. Density plots of cardiac imaging phenotypes calculated from synthetic cardiac meshes conditioned on age and sex. The plots show the distributions of left ventricular mass (LVM), left ventricular end-diastolic volume (LVEDV), left ventricular end-systolic volume (LVESV), right ventricular end-diastolic volume (RVEDV), and right ventricular end-systolic volume (RVESV) derived from synthetic cardiac mesh sequences. For the top row, x-axis denotes age. For the bottom row, x-axis denotes sex (0: women; 1: men).

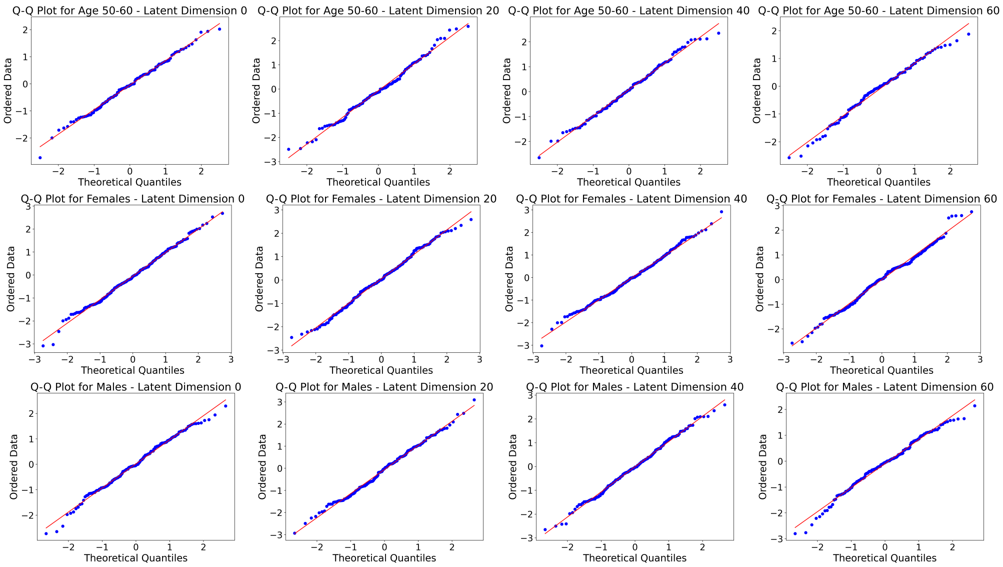

Fig. S3. Q-Q plots to compare one dimension of the latent vector z_a against an ideal Gaussian distribution. The three rows show the plots for the male group (Row 1), female group (Row 2), and 50-60 year-old age group (Row 3). The four columns show four representative latent dimensions, including the 0-th, 20-th, 40-th, and 60-th dimension of the latent vector z_a.

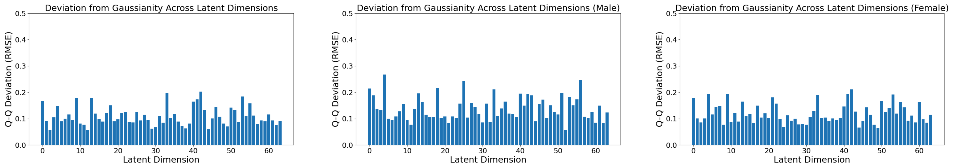

Fig. S4. Deviation from Gaussianity, in terms of the root mean squared error (RMSE) for each of the 64 latent dimensions of z_a.
